# Specialist advice may improve patient selection for decompression therapy following diving accidents: a retrospective observational study

**DOI:** 10.1186/s13049-017-0447-0

**Published:** 2017-10-19

**Authors:** Daniel Steffensmeier, Roland Albrecht, Jürg Wendling, Roger Melliger, Donat R. Spahn, Philipp Stein, Christophe Wyss

**Affiliations:** 1 Institute of Anaesthesiology, University and University Hospital Zürich, Rämistrasse 100, 8091 Zürich, Switzerland; 2 Swiss Air-Ambulance, Rega (Rettungsflugwacht/Guarde Aérienne), Zürich, Switzerland; 3 Divers Alert Network DAN SUISSE, Biel, Switzerland; 4 Melliger analytics AG, Regensdorf, Switzerland; 5 Heart Clinic Zürich, Zürich, Switzerland

**Keywords:** Diving accident, Decompression injury, Air rescue, Pressure chamber, Hyperbaric oxygen

## Abstract

**Background:**

Even in a landlocked country like Switzerland recreational diving is becoming more and more popular. Smaller lakes in the Alps are located at an altitude of 2500 m above sea level. The incidence of diving accidents among all helicopter emergency service missions and the consecutive medical knowledge about decompression injuries is low. Thus, a collaboration between the Swiss Air-Ambulance (Rega) and the divers alert network (DAN) was initiated to improve patient treatment and identification of decompression injury and necessity of hyperbaric oxygen therapy (HBO).

**Methods:**

Retrospective observational study that includes all patients treated by the Rega which have been classified to have had a diving accident from 2005 to 2014. Patient and diving epidemiology was assessed and the impact of DAN collaboration on patient selection and identification of patients needing transport to HBO facilities were analysed.

**Results:**

In the 10-year observational period 116 patients with diving accidents were treated by Rega. Mean patient age was 40 (SD 11) years and 95 (82%) were male. If the Rega emergency physician suspected a decompression injury (DCI), without DAN contact 27/28 (96%) of these patients were transported directly to a HBO facility, whereas with DAN contact only 53/63 (84%) needed transport to a HBO facility. DAN was involved in 66/96 (69%) of the cases with suspected DCI on scene, with a significant increase over time (*p* = 0.001). Mean flight time to HBO facilities was significantly longer (28.9, SD 17.7 min.), compared to non-HBO facilities (7.1, SD 3.2 min., *p* < 0.001). Due to specialist advice, patients may have been selected who finally did not need a transport to a HBO facility, although DCI was primarily suspected by the emergency physician on the scene. These patients experienced a significantly reduced flight time to the (non-HBO) hospital of 25.6 (SD 6.5) min. (p < 0.001).

**Discussion:**

Collaboration of DAN and Rega may allow a safe patient selection and a consecutive reduction of flight time and costs. Due to international collaborations, evacuation to HBO-facilities for acute recompression therapy can be provided by HEMS within less than 30 min all over Switzerland.

**Conclusions:**

For diving accidents among HEMS missions, specialist advice by diving medicine specialists (DAN) appears mandatory to accurately identify and transport patients with decompression injury, as exposure of emergency physicians towards diving accidents and the diagnosis of DCI is low.

## Background

Even in a landlocked country like Switzerland recreational diving is becoming more and more popular. Demographic statistics show that 0.8% of the Swiss population dive [1]. Although diving itself is generally not being described as exhausting [2], stress can vary with changing diving environment (depth, current, temperature) [3–5]. Diving in Switzerland can generally be described as demanding, as the activities take place in cold water mountain lakes, rivers or caves at increased altitude: smaller lakes in the Alps are located at an altitude of 2500 m above sea level. At 4 °C water temperature, dry suit diving is widely-used and depth up to 50 m are very common. Extreme deep diving down to 180 m takes place in a small but active technical diver community [6]. In Switzerland, there are approximately four deadly diving accidents per year [7]. Previously collected data shows that three quarters of the deceased drowned, while nearly 15% died following decompression injury (DCI). In most cases the chain of events could not be fully reconstructed. Related to the 3.6 million economically active citizens of Switzerland, per year 8900 suffer from non-fatal bathing or water accidents. Thereof 1/3 of the accidents happen abroad and 3% (approximately 180) are related to diving [1]. The estimated number of unknown cases is considerably high. The last publicized systematic data survey in Switzerland until now is 35 years old [8].

Rescue attempts with helicopters can be technically and medically challenging, as in-flight hypobaric conditions can lead to exacerbation of symptoms in case of DCI [9]. Recommended maximum flight altitude is 500 ft. (154 m) above starting altitude [10]. In this context, the topographic conditions in Switzerland turn out to be especially challenging: the average altitude above sea level and the Alps may cause a helicopter to take long detours to arrive at destination [11]. It seems likely that between primary care and decompression therapy an undesired delay can often be observed. Keeping this delay as short as possible is crucial as delays in the chain of rescue can be the cause of disabling residual symptoms. Due to decreased availability of decompression chambers in Switzerland in the past years, air transport via helicopter is often necessary as transportation time via road can take hours [6, 10]. By the 1980’s a network of up to ten hyperbaric chambers had been established and Swiss Air-Ambulance (German: Schweizerische Rettungsflugwacht, French: Garde aérienne Suisse de sauvetage, Rega) helicopters were able to take a patient to a nearby decompression chamber in up to 20 min. From 2004, new public health regulations forced hospitals to comply budget regulations and as a result, most of the hyperbaric chambers were shut down or were no longer available for helicopter emergency medical service (HEMS). Today the last operating hyperbaric chamber on Swiss soil is found in the Hôpitaux Universitaires de Gèneve (HUG), in the south-westernmost part of the country [12]. Other decompression chambers now frequently being approached are set in Uberlingen and Ulm in Germany. Other options include Cittiglio and Zingonia (Bergamo) in Italy for diving accidents that have taken place south of the Alps. Not only has this resulted in an increased treatment delay but also to a longer occupation time for the transporting helicopters, making them unavailable for a longer period of time [6].

Rega collaborates with the Divers Alert Network (DAN) which is a worldwide non-profit organization that offers 24/7 emergency medical advice for diving accidents. The Swiss medical team is composed of diving medicine physicians trained by the European Diving Technical Committee (EDTC) or the Diving Medical Advisory Committee (DMAC) and gives telemedical assistance for diving emergencies in Switzerland and abroad. The physician on call gathers information about the accident history and current symptoms and decides whether there is an indication for immediate recompression (HBO therapy) or not. If HBO therapy is necessary Rega will organize the treatment and transfer of the patient to the next available decompression chamber [12].

The objective of this study is to analyse all patients with diving accidents and their topographic distribution in Switzerland from 2005 to 2014, who were transported by the HEMS Rega. Information on diving and patient epidemiology will be presented. The impact of Rega and DAN collaboration on patient selection and transport time will be analysed with the hypothesis that DAN involvement may improve patient selection for HBO therapy.

## Methods

### Ethics

The data analysis was approved by the local ethics committee (Kantonale Ethikkommission Zürich, Switzerland, KEK-ZH 2015–0194).

### Study design and participants

Retrospective observational study that includes all patients treated by the Swiss Air-Ambulance (Rega) which have been classified to have had a diving accident from 2005 to 2014. Data collection, interpretation and manuscript preparation is in accordance with the Strengthening the Reporting of Observational Studies in Epidemiology (STROBE) guidelines [13, 14].

### Setting

As described by Schmidt et al. [15], Rega is a non-profit HEMS that performs more than 11.000 emergency missions per year from 12 bases and one partner-base in Switzerland (except for the Canton Valais). Operation profiles comprise primary (scene to hospital) and secondary (inter-hospital transport) missions with all type of emergencies (medical, trauma, evacuations) and patient characteristics. The HEMS Crew consists of a helicopter pilot, a paramedic and a specially trained emergency physician. The HEMS operates during day- and night-time. For patients included to this analysis, Rega HEMS received the emergency call directly or in parallel to the DAN network. It was in the discretion of the emergency physician to involve DAN, if required.

### Definitions

#### Decompression injury (DCI)

The mechanism of DCI is the formation of gas bubbles in body tissue which leads to clotting of capillaries and thus to hypoxia of the affected area according to Henry’s Law. The effects of dissolved gas expansion range from asymptomatic bubbles to spinal or cerebral involvement that can result in paraplegia, seizures or death [9]. The goal of decompression stops while ascending is to limit the formation of gas vesicles by reducing the partial pressure difference of the inert gases so that there is enough time to eliminate micro vesicles via the blood stream and the lungs [16]. The Rega emergency physician defined if, or if not decompression injury may have been a plausible and explainable mechanism, accounting for the diver’s accident. This binary variable distinguishes drowning and other obvious causes not related to diving from possible decompression injuries (upon judgement by the Rega physician).

#### DCI-classification

DCI classification is not trivial, as clinical symptoms may resemble different entities of decompression injury, especially if judged in the emergency setting. Decompression sickness (DCS, type 1, mild and type 2, severe) excludes the separate diagnosis of arterial gas embolism. Severe DCS may lead to gas embolization to the systemic circulation without concomitant lung injury. Clinical symptoms on the scene of accident are not sufficient to establish a definitive diagnosis in regard to the type of decompression injury and or arterial gas embolism. We therefore classify the symptoms described by the emergency physician towards light (skin and joint involvement), severe (neurological dysfunction, inner ear involvement) and ambiguous or non-classifiable symptoms (n/c).

### Descriptive variables

Over the course of ten years the operating base, emergency location, destination, flight minutes/occupancy of the helicopter, time and season as well as age and gender of the patients have been recorded in the Rega database. Rega and DAN databases were compared to evaluate collaboration of the two organizations. Patient’s vital signs, performed medical procedures (e.g. intubation, resuscitation) as well as the physicians suspected diagnosis (DCI yes/no) and presumed DCI category were extracted from the Rega medical records. If possible, we also collected diving-related variables, such as duration of the dive and maximum depth. If DAN was contacted by the crew on site, information about the necessity of decompression therapy and intensive care treatment was available.

#### Endpoints and outcome variables

1. Description of patient and diving accident epidemiology in Switzerland during the study period.
2. Analysis of the impact of Rega and DAN collaboration on patient selection and flight time in the context of a changed HPC landscape
3. Topographic distribution of the accident sites in regard to transport and treatment facilities.

#### Data collection

Relevant missions were identified in the Rega database and characteristics were extracted. From the protocols written by the treating emergency physicians on site, medical treatment as well as the patient’s symptoms were recorded. The diving accident was characterized according to the suspected DCI type. To analyse the collaboration between Rega and DAN over course of time, the two databases were compared.

#### Bias

The Rega database contains every helicopter movement, as obliged by law. These movements are linked to mandatory information on mission and patient characteristics limiting the influence of selection bias. The analysed HEMS protocols were completed directly at the end of every mission by the responsible emergency physician, narrowing the effect of a recall bias [15]. Data was available for patients transported by Rega HEMS only. Epidemiology data from this analysis cannot be generalized for all diving accidents, as patient selection may have been in favour of severely affected patients.

### Statistic

Statistical data was analysed with the SPSS software (Version 24, IBM, Armonk NY, USA). Categorical data was presented as frequency and percent, numerical data was presented as mean and standard deviation (SD) and [minimum-maximum]. For numerical variables we used the “Wilcoxon-Mann–Whitney” test. Chi square test was used to test independence of categorical data. A two-sided p-value of less than 0.05 was considered statistically significant.

## Results

### Patient and diving characteristics

In the 10-year observational period 116 patients with diving accidents were treated by Rega. Mean patient age was 40 (SD 11), [15–80] years and 95 (82%) were male. Mean diving depth was 44 (SD 25), [3–190] meters and mean duration of dive was 36 (SD 35), [6–240] minutes. Ninety-six patients (83%) were judged as DCI by the treating emergency physician on scene. DCI types are summarized in Table 1. Nineteen patients (17%) received on scene resuscitation, 6 of them could be transported after successful primary resuscitation and 13 were declared dead on scene. Of the HEMS treated patients, 80/116 (69%) were directly referred to a centre with HBO capability. Of all missions, 37 (32%) were performed at night time. No exacerbation of DCI symptoms during in-flight hypobaric conditions have been reported.

**Table 1:** Patient and mission epidemiology for Rega diving accident missions

		2005 (*n* = 11)	2006 (*n* = 10)	2007 (*n* = 16)	2008 (*n* = 12)	2009 (*n* = 15)	2010 (*n* = 12)	2011 (*n* = 11)	2012 (*n* = 13)	2013 (*n* = 10)	2014 (*n* = 6)	Total (*n* = 116)
Age (years)		41 (8)	30 (10)	35 (7)	44 (7)	42 (11)	41 (12)	39 (13)	39 (11)	52 (13)	35 (8)	40 (11)
Gender (male)		10 (91%)	8 (80%)	12 (75%)	10 (83%)	10 (67%)	11 (92%)	9 (82%)	9 (69%)	10 (100%)	6 (100%)	95 (82%)
Heart rate (1/min.)		63 (35)	88 (18)	79 (15)	90 (20)	91 (18)	82 (19)	81 (13)	88 (13)	100 (30)	95 (9)	85 (22)
Systolic blood pressure (mmHg)		114 (51)	135 (16)	127 (25)	140 (10)	120 (17)	130 (29)	141 (15)	123 (9)	130 (26)	112 (10)	127 (24)
Diving depth (m)		26 (14)	44 (14)	44 (26)	38 (9)	51 (48)	40 (15)	40 (7)	36 (11)	51 (25)	69 (36)	44 (25)
Diving time (min.)		34 (7)	20 (7)	22 (21)	27 (15)	40 (46)	45 (11)	29 (27)	32 (17)	29 (10)	89 (102)	36 (35)
On site intubation		0	1 (10%)	4 (25%)	2 (17%)	3 (20%)	1 (8%)	2 (18%)	2 (15%)	2 (20%)	0	17 (15%)
On site CPR		2 (18%)	1 (10%)	3 (19%)	2 (17%)	3 (21%)	1 (8%)	2 (18%)	2 (15%)	3 (30%)	0	19 (17%)
DCI suspected by emergency physician	% of diving accident	9/11 (82%)	9/10 (90%)	14/16 (88%)	10/12 (83%)	12/15 (80%)	10/12 (83%)	7/11 (64%)	10/13 (77%)	9/10 (90%)	6/6 (100%)	96/116 (83%)
DCI type suspected by emergency physician	light	0	3	1	2	1	0	1	0	0	0	
	severe	8	6	10	8	9	6	5	9	8	6	
	n/c	1	0	3	0	2	4	1	1	1	0	
DAN contact	% of DCI	3/9 (33%)	5/9 (56%)	7/14 (50%)	7/10 (70%)	8/12 (67%)	9/10 (90%)	5/7 (71%)	8/10 (80%)	8/9 (89%)	6/6 (100%)	66/96 (69%)
Transport to HBO facility	% of DCI	7/9 (78%)	7/9 (78%)	12/14 (86%)	8/10 (80%)	9/12 (75%)	9/10 (90%)	7/7 (100%)	9/10 (90%)	7/9 (78%)	5/6 (83%)	80/96 (83%)
	with DAN contact	3/7 (43%)	4/7 (57%)	5/12 (42%)	5/8 (63%)	5/9 (56%)	8/9 (89%)	5/7 (71%)	7/9 (78%)	6/7 (86%)	5/5 (100%)	53/80 (66%)
	without DAN contact	4/7 (57%)	3/7 (43%)	7/12 (58%)	3/8 (37%)	4/9 (44%)	1/9 (11%)	2/7 (29%)	2/9 (22%)	1/7 (14%)	0	27/80 (44%)

Patient, diving and emergency treatment epidemiology per year (2005–2014) and total. Numbers are mean (standard deviation) and n (%). CPR: cardiopulmonary resuscitation, HBO: hyperbaric oxygen, DCI: decompression injury

### Rega / DAN collaboration

**Fig. 1:**
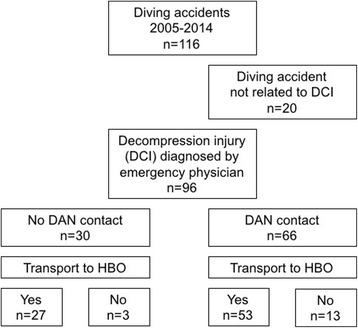
Flowchart. Diving accidents in Switzerland treated by Swiss HEMS Rega. Five patients with suspected DCI died on the scene and were thus not transported to a hospital (3 with DAN contact and 2 without DAN contact). HBO: hyperbaric oxygen, DCI: decompression injury

**Fig. 2:**
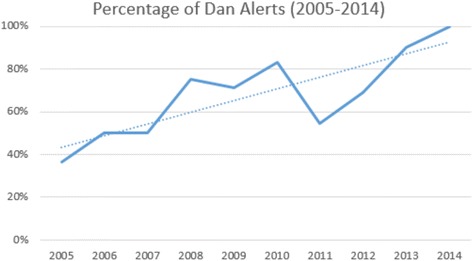
Percentage of DAN alerts over time. Significant increase of DAN contact in the case of a diving accident treated by the Swiss HEMS Rega

DAN was involved in 66/96 (69%) of the cases with suspected DCI on scene, with a significant increase over time (Figs. 1 and 2, Table 1, *p* = 0.001). If the on-scene emergency physician suspected a DCI, without DAN contact 27/28 (96%) of these patients were transported directly to a HBO facility, whereas with DAN contact only 53/63 (84%) were transported to a HBO facility. Five patients with suspected DCI died on the scene and were not transported to a hospital (3 with DAN contact and 2 without DAN contact). None of the investigated patients needed a secondary transfer to an HBO facility after initial HEMS transport to a non-HBO facility.

### Treatment and transport time

**Fig. 3:**
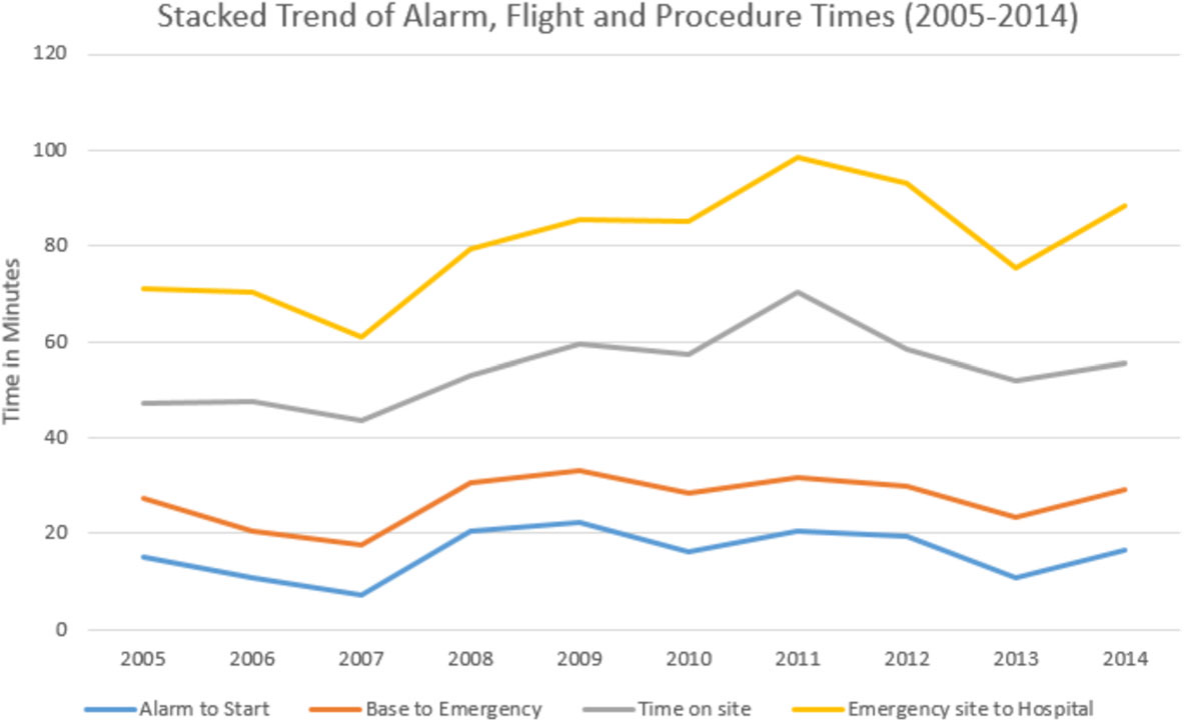
Swiss HEMS Rega flight times over time. Flight times for Rega HEMS missions in the case of a diving accident in Switzerland. Blue line: Time from alert to start, orange line: Time from start to the scene, grey line: time at the scene, yellow line: flight time from the scene to the hospital

Mean flight time to the scene was 11.2 (SD 5.7) minutes, mean time on scene was 27.4 (SD 15.6) minutes, and mean flight time from the scene to the hospital was 25.7 (SD 18.1) minutes. Flight times did not differ significantly over time (Fig. 3). Mean flight time to HBO facilities was significantly longer (28.9, SD 17.7 min.), compared to non-HBO facilities (7.1, SD 3.2 min., *p* < 0.001). Compared to overall general primary Rega missions, diving accident missions show comparable flight time to the scene (11.3, SD 1.1 min., vs. 11.4, SD 0.3 min., *p* = 0.73) but significantly longer flight time from the scene to the hospital (26.3, SD 5.0 min. vs. 9.7 SD 0.4 min., *p* < 0.001). Due to specialist advice, patients may have been selected, who finally did not need a transport to a HBO facility, although DCI was primarily suspected by the emergency physician on the scene. This advice resulted in a transport to a non-HBO hospital with significantly shorter flight time of 25.6 (SD 6.5) min. (*p* < 0.001) compared to patients needing transport to HBO facilities.

### Topographic distribution

**Fig. 4:**
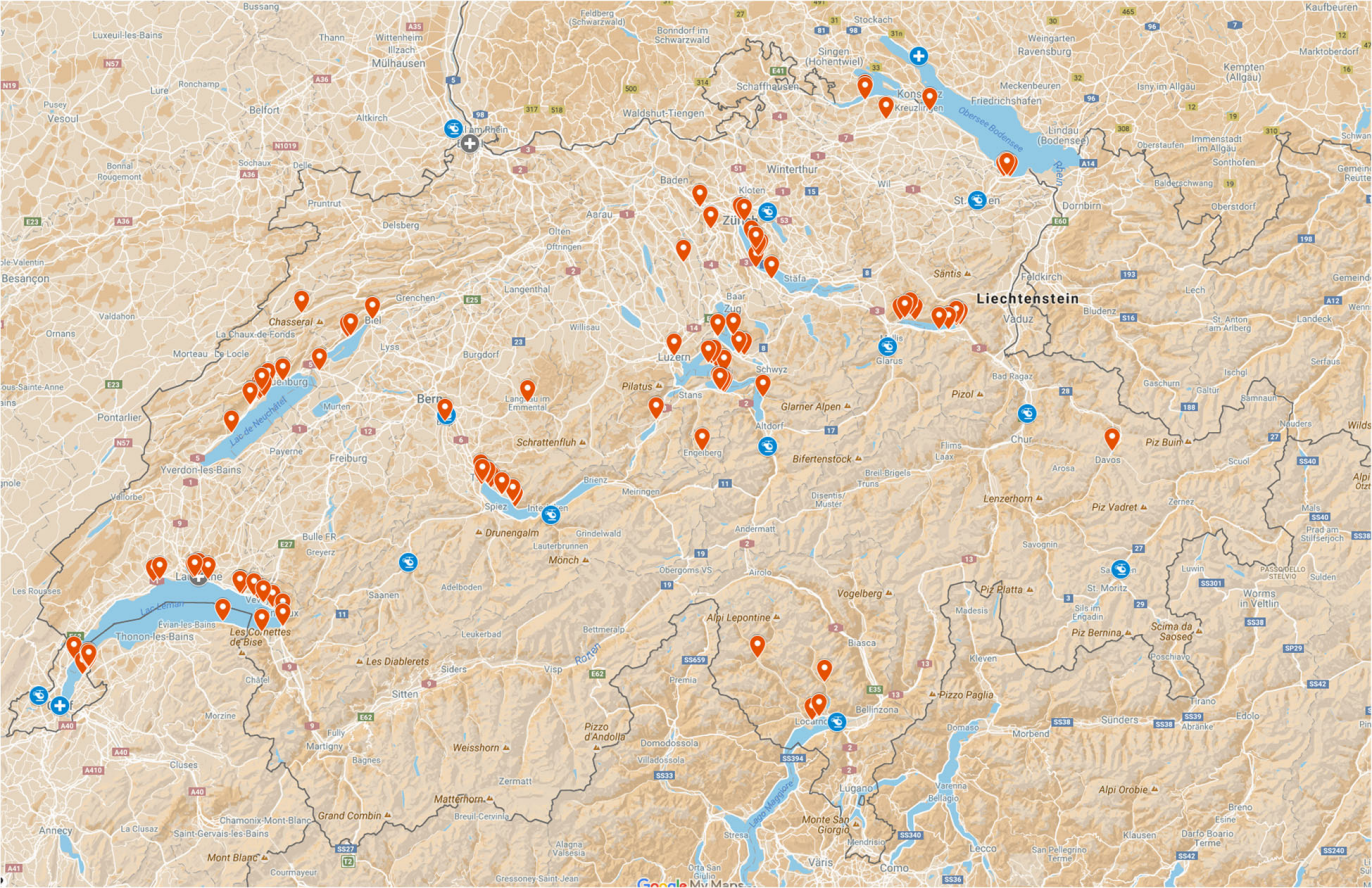
Geographical distribution of diving accidents in Switzerland. All 116 patients or diving accident sites are depicted (red balloons). HBO facilities are (+) within blue (active) and grey (inactive) points. Distribution of Rega HEMs helicopter bases throughout Switzerland (white helicopter within blue point). Mapdata by Google © 2016 GeoBasis-DE/BKG (©2009), Google, Inst. Geogr. Nacional

Except for the accidents in the Canton Ticino, the vast majority of the accident sites was located north of the Alps or within the Alps with the possibility to reach the next decompression facility with a helicopter flight at low altitude or with a general descent. Helicopter bases are located within a flight time of 15 min or less around the diving accident sites. The accident sites are almost equally distributed to the eastern and western part of Switzerland. The closest available decompression chambers are thus Uberlingen (Germany) and Ulm (Germany) for accidents in the eastern part, Geneva is the closest decompression chamber for accidents in the western part of Switzerland (Fig. 4).

## Discussion

Epidemiological data on diving accidents in Switzerland are scare and the estimated number of unknown cases is considerably high. The latest systematic data survey was published in 1981 [8]. In the present study, we characterized systematically all Swiss Air-Ambulance (Rega)-missions on 116 patients classified to have had a diving accident from 2005 to 2014. This analysis does not represent a general survey on diving accidents in Switzerland, since the need of HEMS-evacuation is a substantial selection bias. Related to a Swiss water accident analysis 10–16 of approximately 180 non-fatal and 4 fatal diving accidents per year are primarily transported by Swiss HEMS Rega. Most of the patients in this study suffered from advanced decompression injury (high prevalence of severe DCI, 17% received resuscitation on scene, 11% acute mortality, 63–83% had been evacuated to HBO-facilities).

Tactical HEMS-evacuation situation for diving accidents in Switzerland is currently influenced by limited HBO-facilities in range (only one in Switzerland, 2–3 in the nearby neighbour countries) and the specific topographic conditions (high average altitude above sea level and the Alps that may cause a helicopter to take long detours to arrive at destination) [11]. In our study population, no exacerbations of DCI symptoms during in-flight hypobaric conditions were reported. Compared to overall general primary Rega missions, diving accident missions show comparable flight time to the scene but significantly longer (more than doubled) flight time from the scene to the target hospital (increased logistic effort).

Interestingly, almost one third of all diving accident missions are night missions, which makes them technically demanding. This fact could be explained by long flight times to the specialized target hospitals in combination with local diving habits of rather private after-work leisure diving in the late afternoon than commercial morning/day trips like in other countries (no relevant diving tourism in Switzerland).

Topographic distribution of diving accidents is equal around almost all lakes of the populated areas all-over Switzerland, no specific “hot-spots” could be identified. No clear trends in seasonal distribution were observed in the present study, diving accidents occur at any time of the year in Switzerland.

Since diving accidents are rare in general and need specific knowledge in DCI-diagnosis and treatment, telemedical counselling of the HEMS-physician by diving medicine specialists (e.g. DAN) can be beneficial for patient triage. The collaboration between Rega and DAN tightened in the studied time period, reflected by a significant increase in direct DAN-involvement in suspected DCI over time (27% DAN-contact in 2005, 100% in 2014). This resulted in improved patient selection for acute HBO-therapy and consecutive reduced mean flight times for diving accident missions. Furthermore, no patient needed a secondary transfer to an HBO facility after DAN contact. This implies a safe practice of patient selection by DAN specialist advice and allows tailored treatment and transfer of patients suffering from diving accidents and DCI. Moreover, this selection leads to a significant reduction of flight time and thus a significant cost reduction for the patient and the insurance company respectively.

Limitations: Due to the retrospective design, conclusions may not be applicable in different settings and causal relationship for the outcome variables cannot be inferred. Flight time and the Swiss setting may be of special local or HEMS interest only. It may be possible that the patient selection for transport destination after DAN advice was confounded by indication.

## Conclusions

HEMS-missions for diving accidents in Switzerland are rare but logistically, technically and medically demanding. Collaboration of DAN and Rega may allow a safe patient selection and a consecutive reduction of flight time and costs. Due to international collaborations, evacuation to HBO-facilities for acute recompression therapy can be provided by HEMS within less than 30 min all over Switzerland in a substantial part of the cases where ground transportation would not be achievable within this time frame. For diving accidents among HEMS missions, specialist advice by diving medicine specialists (DAN) appears mandatory to accurately identify and transport patients with decompression injury, as exposure of emergency physicians towards diving accidents and the diagnosis of DCI is low.

## Abbreviations

DAN: Divers alert network
DCI: Decompression injury
HBO: Hyperbaric oxygen
HEMS: Helicopter emergency medical service
Rega: Swiss Air-Ambulance, Rega (Rettungsflugwacht/Guarde Aérienne)
